# Food environment interactions after migration: a scoping review on low- and middle-income country immigrants in high-income countries

**DOI:** 10.1017/S1368980021003943

**Published:** 2022-01

**Authors:** Aravinda Berggreen-Clausen, Sai Hseing Pha, Helle Mölsted Alvesson, Agneta Andersson, Meena Daivadanam

**Affiliations:** 1 Department of Food Studies, Nutrition and Dietetics, Uppsala University, Husargatan 3, Box 560, Uppsala 75122, Sweden; 2 Department of Global Public Health, Karolinska Institutet, Solna, Stockholm, Sweden; 3 International Child Health and Nutrition Research Group, Department of Women’s and Children’s Health, Uppsala University, Uppsala, Sweden

**Keywords:** Scoping review, Food environment, Migration, Food access, ANGELO framework, Food choice, Immigrants

## Abstract

**Objective:**

To map and characterise the interactions between the food environment and immigrant populations from low- and middle-income countries living in high-income countries.

**Design:**

A scoping review was carried out following the framework outlined by Arksey and O’Malley, as well as Levac *et al.* Peer-reviewed studies in English published between 2007 and 2021 were included. Two reviewers screened and selected the papers according to predefined inclusion criteria and reporting of results follows the PRISMA-ScR guidelines. A ‘Best fit’ framework synthesis was carried out using the Analysis Grid for Environments Linked to Obesity (ANGELO) framework.

**Setting:**

High-income countries.

**Participants:**

Immigrants from low- and middle-income countries.

**Results:**

A total of sixty-eight articles were included, primarily based in the USA, as well as Canada, Australia and Europe, with immigrants originating from five regions of the globe. The analysis identified three overarching themes that interconnected different aspects of the food environment in addition to the four themes of the ANGELO framework. They demonstrate that in valuing fresh, healthy and traditional foods, immigrants were compelled to surpass barriers in order to acquire these, though children’s demands, low incomes, time scarcity and mobility influenced the healthiness of the foods acquired.

**Conclusion:**

This study brought together evidence on interactions between immigrant populations and the food environment. Immigrants attempted to access fresh, traditional, healthier food, though they faced structural and family-level barriers that impacted the healthiness of the food they acquired. Understanding the food environment and interactions therein is key to proposing interventions and policies that can potentially impact the most vulnerable.

## Graphical abstract

**Figure:**
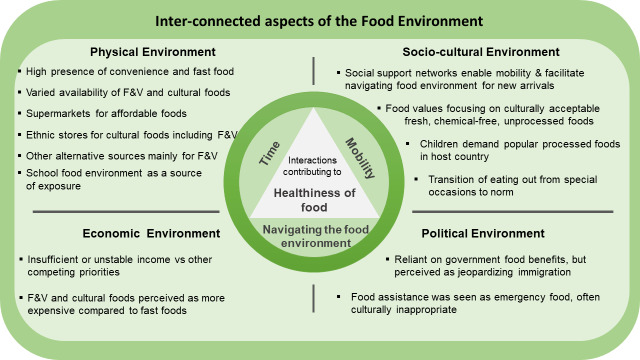

Migration to high-income countries is linked to increased risk of non-communicable diseases ^(1)^. Immigrants from low- and middle-income countries moving to high-income countries experience an abrupt change from a more traditional food environment to a modern, industrialised one^(2)^ and over time they suffer from higher rates of many negative health outcomes, including obesity, type 2 diabetes and other diet- and metabolism-related chronic diseases, often at a younger age^(1,3,4)^. Immigrants have reported abrupt changes in the social and environmental structure including lack of time, lack of social relations, more stress, children’s preferences, taste, food insecurity and lack of access to traditional foods leading to having a less healthy lifestyle after migrating^(5,6)^.

The food environment can be considered as the interface between the food system and consumers’ food acquisition^(7)^. Availability of unhealthy foods has been linked to obesity more consistently than availability of healthy foods^(8)^ as observed through the presence of energy-dense nutrient-poor foods^(9,10)^ and greater access to unhealthy food outlets^(11)^; this availability has also been linked to type 2 diabetes^(12)^. Accessing foods is a complex dynamic of availability, accessibility and social, cultural and material conditions^(13,14)^. Additionally, perceived access has been found to relate more to dietary behaviour than to objective measures such as distance to stores^(13–15)^. Residing in socioeconomically disadvantaged neighbourhoods has been consistently associated with obesogenic dietary behaviour and higher rates of diet-related non-communicable diseases^(11,16)^. However, living in a neighbourhood with a high immigrant density has been found to be protective against the dietary changes acquired through acculturation^(17)^.

How interactions take place with the food environment are not well understood, specifically the interactions between immigrants, the food environment in the host countries and their potential impact on acquisition of food. An important step in characterising these interactions is to synthesise what is known about immigrants and their food environment. A scoping review was therefore conducted with the aim to map and characterise the interactions between the food environment and immigrant populations from low- and middle-income countries living in high-income countries, as well as identify research gaps.

## Methods

A scoping review protocol was developed and revised by the research team, and the final protocol was registered with the Open Science Framework on 29 January 2021 (https://osf.io/vzx57). We performed the scoping review using the methodological framework outlined by Arksey and O’Malley^(18)^ and further developed by Levac *et al.*^(19)^. The review followed five key phases: (1) identifying the research question; (2) identifying relevant studies; (3) study selection; (4) charting the data and (5) collating, summarising and reporting the results. The reporting is described as per the PRISMA-ScR guidelines^(20)^.

### Identifying relevant studies and study selection

Relevant literature on the food environment and immigrants in high-income countries published in English between 1 January 2007 and 14 May 2021 was eligible for inclusion, the latter being the date of the last search. Grey literature was subsequently not included since initial searches and reading found negligible grey literature on the subject. Three electronic databases (EMBASE, PubMed and Web of Science) were used as primary search sources. A search strategy was developed in consultation with a librarian through Uppsala University Library. Key words relevant to food environment and immigrant population were formed into a search string (see online Supplemental Table 1). Backward and forward snowballing and hand searches were performed to identify additional articles.

Two reviewers carried out the initial search and used the Rayyan QCRI for independently screening titles and abstracts^(21)^. At the title and abstract screening phase, only articles related to migration from low- and middle-income countries to high-income countries, coupled with aspects relating to the food environment, were included (see online Supplemental Table 2). The full-text screening was also performed by two reviewers; a simple data collection form was developed to assess the relevance of the articles in order to facilitate consistency in the inclusion and exclusion process. Disagreements on study selection were resolved by consensus and discussion and when necessary, consulted the third reviewer. No critical appraisal was carried out on the studies as scoping reviews do not usually include this step^(22)^.

### Charting the data

In this stage each of the eixty-eight articles were read thoroughly, followed by charting of the data extracted based on the PICO model (population, intervention, comparison, outcome of interest) (see Table 1). Each of the two reviewers charted half of the articles and reviewed one another’s charting.

**Table 1:** Charting form of included articles

Author/s, year	Objective	Duration in host country	Study setting; host country	Country of origin/sample characteristics; (*n*); sampling methods	Study design and methods	Key outcome contributed to the study
Addo *et al.*, 2019^(80)^	To examine the dietary and physical activity behaviours of sub-Saharan African population	Less than 10 years (*n* 14); more than 10 years (*n* 10)	2 states, New South Wales and Victoria, Australia	Sub-Saharan African (*n* 24); (urban); quota sampling	Qualitative; Phenomenological approach; Semi-structured interviews	In Australia, high cost of local African foods; easy accessibility of fast and processed foods; limited availability or accessibility of some African foods, leading to use of host country foods. Home country foods are perceived as more natural, Australian foods of lower quality. Lack of trust towards host country foods due to not knowing the source of foods. Less time available to cook led to more convenience foods.
Amos and Lordly 2014^(70)^	To explore international students’ Canadian food experience through the use of photovoice	Not specified. university students	Mount Saint Vincent University, Canada	International students (*n* 15); (university); convenience sampling	Qualitative; photovoice approach	High availability of foods generally in host country. The paradox of Canadian convenience (easy but with negative impact on health). Traditional and organic foods perceived as healthy and were preferred. Food quality and accessibility were important factors in food choices. Able to access traditional food, though it tasted better in home country. Support networks through food. Exploration of non-traditional foods in Canadian food culture. Eating out at ethnic restaurants.
Amstutz*et al*., 2020^(90)^	To investigate the prevalence of undernutrition and obesity among refugees in Geneva and to identify barriers to healthy eating.	18 months	Geneva, Switzerland	(68 % were men, mainly from Eritrea, Afghanistan, Sri Lanka and Syria), anthropometric survey (*n* 354 adult refugees), seven FGD (*n* 51); purposive sampling	Mixed methods; qualitative (FGD), quantitative	Affordability: the main problem was the high cost of healthy foods. Lack of cooking skills and the need to acquire new culinary skills. They valued natural unprocessed foods, without chemicals. Prioritised quantity over quality due to low incomes. Language barrier hindered understanding food labels and buying traditional food. Facilitators for eating healthier were health problems that required dietary change; financial and housing autonomy to manage one’s budget and cook oneself. Personal food preferences for high-energetic foods was a barrier to eating healthier.
Blanchet *et al.*, 2017^(72)^	To explore immigrant children’s lived experience of dietary acculturation	About 10 years (10·6 years; sd, 9·1 years)	Ottawa city, Canada	Sub-Saharan African and the Caribbean (*n* 167); (urban); non-probability sampling	Qualitative; draw and tell	Convenience and accessibility to fast foods, lack of time and energy led to usage. Most children did not bring traditional food as school lunch. Children liked Canadian food and parents liked traditional food.
Blanchet *et al.*, 2018^(71)^	To explore the process of dietary acculturation among black immigrant families of African and Caribbean descent	Less than 10 years (*n* 7); more than 10 years (*n* 5)	Ottawa city, Canada	Sub-Saharan African and the Caribbean (*n* 12); (urban)	Qualitative; in-depth interviews	Low access to quality affordable traditional food, high access to cheap processed Canadian food. Lack of time led to shortcuts. Children exposed to host country foods through school. Children influenced food at home through demands and refusals.
Bojorquez *et al*., 2018^(25)^	To describe the changes in dietary practices of Mexican women, return migrants from USA	Not specified.	Tijuana (border city), USA and Mexico (return migrants from USA)	Mexican migrants return from USA (*n* 27); (urban); purposive sampling	Qualitative; social practice framework; semi-structured interviews	The increase in frequency of fast food and ready meals with time constraints, limitations to food access, modifications in food-purchasing practices related to taste, quality and health aspects. Women working, not having time and lack of support. The influence of socio-economic situation on priorities and values relating to food. Perceived traditional food as ‘good food’. Those from urban areas more familiar with Western foods compared with from rural areas.
Bowen *et al.*, 2019^(26)^	To examine factors affecting the food choices of Latino immigrant mushroom farm workers and their families	Seasonal workers (temporary residency 3 years)	Pennsylvania, USA	Latino & non-Latino (*n* 111); (rural); convenience sampling	Quantitative;cross-sectional study	Cost as a barrier to healthy food choices, not knowing how to cook healthy food, highly influenced by what family would eat. Access to fresh vegetables largely through food bank. Healthy food choices dependent on availability.
Burge and Dharod, 2018^(27)^	To assess food choices, shopping and spending practices among the Southeast Asian refugee group of “Montagnard” resettled in the USA	Had lived in the USA on average 6 years	USA	Montagnard refugee women (*n* 12); Convenience and snowball sampling	Quantitative; grocery food receipt analyses	Visited several stores for acquiring food, both from regular stores and ethnic stores. Distance, familiarity with the food choices and relationships with owners in ethnic stores were important determinants of store choice. Food purchases using the SNAP benefits used up quickly, bulk buying of staples to last the month. Cultivated own food to save money.
Carney and Krause, 2020^(55)^	To examine the possibilities for engaged research oriented towards generating “healthy publics” and addressing food insecurity across disparate geo- graphical and political settings and amid structural and social constraints	Not clear; (3 months to 30 years)	1. Santa Barbara County, California, USA 2. Rural Haiti, Dominican Republic 3. Sicily, Italy	1. Mexican and Central American (*n* 25); 2. Haitian and Dominican (*n* 60); 3. African immigrants	Mixed methods: ethnographic fieldwork and participant observation, a cross-sectional occupational health survey and semi-structured qualitative interviews	Affordability: making it work, reducing the amount eaten, relying more on ‘basics’ like potatoes, tortilla, beans, finding cheaper options. Availability: quantity is sufficient but not the quality (less money/too expensive). Accessibility: lack of car, hours of investment going from store to store/reading coupons, scared to access government food assistance due to being undocumented
Cerin *et al*., 2019^(81)^	To examine built and social environmental facilitators of and barriers to regular engagement in physical activity, eating a healthy diet and regular contact with other people	Less than 5 years (*n* 41); 5–14 years (*n* 18); more than 14 years (*n* 32)	Melbourne, Australia	Mandarin or Cantonese (*n* 91); (urban); purposive convenience sampling	Qualitative; nominal group technique	Cost of foods is a barrier to healthy eating (participants were elders financially dependent on adult children). Their children determined what was eaten at home. Poor/inadequate public transportation, high food safety standards/regulations were considered important to access healthy food. High prevalence of unhealthy options in the food environment.
Chaufan *et al*., 2011^(29)^	To examines multiple social determinants of health, with a focus on disparities in the structural sources of T2DM risk, especially food environments	Not specified.	Northern California, USA	Fifteen Latino clients and six NGO staff (*n* 21); (urban); purposive convenience sampling	Mixed methods: quantitative and qualitative	Local food stores offered a limited variety of healthy foods, at unaffordable prices. Lack of own transport and long distances on public transport were barriers to access, coupled with low incomes and insecure employment. Language barriers and lack of time due to long hours of work and multiple jobs compounded this. There was a high reliance on food assistance.
Chaufan *et al.*, 2012^(28)^	To explore the social determinants of diabetes in a low-income Latino and immigrant neighbourhood, emphasising food environments	Not specified.	Northern California, USA	Fifteen Latino clients and six NGO staff (*n* 21); (urban); convenience sampling	Qualitative; focus group and a semi-structured interviews	High cost of healthy food, low cost of processed foods and low incomes. Lack of time and competing basic needs/constraints of poverty coupled with lack of own transportation, inconvenient public transport made it harder to access healthy food. Language barriers. Insufficient food benefits and assistance; immigration status and stigma were barriers to access.
Colón- Ramos *et al.*, 2017^(30)^	To understand what makes it easier or harder for Latina mothers to provide their children with healthy food	Had lived in the USA for <15 years	Washington, D.C., USA	Latina mothers (*n* 15); (urban); purposive sampling	Qualitative; photovoice approach	Availability and affordability of sugary beverages in the neighbourhood. Low incomes and competing needs. Mothers navigated the neighbourhood food retail environment and decided where to shop based on availability, pricing and quality. Traditional food was valued and considered healthier. Children’s food preferences influenced home food.
Cordeiro *et al.*, 2018^(53)^	To examine the broad issues of food security and access to healthful, cultural food in the context of safety net participation	Not specified.	Lowell, MA, USA	Low-income Cambodians and Brazilians (*n* 84); (urban)	Qualitative: (community-based participatory research and focus groups)	Accessibility: access to healthy, cultural foods but require trips to multiple stores to get the variety desired at affordable prices. Imported cultural foods: expensive and concerns with the quality. Valued healthy cultural foods. Reliance on food assistance differed between the groups, from none to high.
Dawson-Hahn *et al.*, 2020^(31)^	To explore perspectives on nutrition, health and physical activity among immigrant parents with young children before and after migration	Unclear.	Seattle, USA	Five groups (Arabic, Somali, Dari, Burmese and Nepali) (*n* 50); (refugee; urban)	Qualitative; phenomenological approach; focus groups and semi-structured interviews	A difference in access to fresh foods after migration, families from refugee camps reported greater access to fresh foods, organic foods preferred, but expensive.
Dubowitz *et al*., 2007^(32)^	To investigate how life course, immigrant status, acculturation and neighbourhood of residence influence food purchasing and preparation	Not specified.	Massachusetts, USA	Low-income immigrant women (*n* 44); (urban); purposive sampling	Qualitative; focus groups	Physical access to food purchasing points did not influence food purchasing and preparation; price as a key factor in choice of shopping locations as well as challenges in transportation to stores and childcare. Time scarcity meant less time for food shopping. Foods in home country were considered fresher, tastier and better than host country foods that were perceived as old, preserved and contained chemicals.
Evans *et al*., 2015^(54)^	To gather low- income community members’ opinions about their food purchasing choices and their perceptions of the most effective ways to increase access to healthful foods in their communities	Not specified.	Central Texas, USA	Low-income, ethnically diverse communities (*n* 148); (urban); a random sampling	Qualitative: focus groups	Accessibility: long distance to supermarket or large grocery stores. Affordability: high cost of fruits and vegetables relative to low household incomes. Preference for healthier foods.
Fish *et al*., 2015^(56)^	To identify factors influencing fruit and vegetable shopping and use of alternative healthy food options	0–10 years (*n* 14); 10–20 years (*n* 10)	Forsyth County, USA	Latina, low-income (*n* 24); (urban); purposive sampling	Qualitative: face-to-face, in-depth interviews (semi-structured)	Acquired foods from large stores; some shopped from one store, others visited several. Shopping habits determined by proximity of stores, perception of stores and prices. Latinas preferred fresh fruits and vegetables compared with canned/frozen and perceived them as being more nutritious and suited their cooking methods. Not willing to try new foods. Neighbours with gardens shared fresh produce.
Franzen and Smith, 2010^(51)^	To investigate influences on shopping and eating behaviour of Hmong adults living in St. Paul/Minneapolis, Minnesota	1–5 years (*n* 19); 5 years as a marker	Minnesota, USA	Hmong (*n* 69); (urban)	Mixed methods; a mapping project, food surveys, FFQ & focus groups	Store choice: depended on price, food availability and familiarity, American stores for general items, Hmong/Asian for specific items. Use farmers markets during summer months. Drove or took bus to stores.
Fraser*et al*., 2012^(89)^	To measure access to all food outlets in part of a northern UK city and investigate the relationship with body weight, obesity and small area-based deprivation in a multi-ethnic population	Not specified.	Bradford, Metropolitan, UK	Non/South Asian (*n* 1198); (urban)	Mixed methods: GIS mapping and cohort data	South Asians had closer to all food outlets, providing healthy and unhealthy options. Food access measures with borderline significance (specialist shops in ‘super output area’, within 500 m of residence, within 250 m of residence) all negatively associated with BMI.
Fuster and Colón-Ramos, 2018^(33)^	To identify the reinforcing influence of the various environments on individual behaviours	Had been in the USA for <15 years	El Salvador, Central America and Washington D.C., USA	Salvadoran, (*n* 28); (Border/rural); & In US (*n* 30); (Suburb); Purposive sampling	Qualitative: social ecological framework; focus groups and semi-structured interviews	Food environments (schools, food stores) exposed families to highly processed, unhealthy foods. Traditional home-made foods were valued and considered healthy. Children’s preferences for processed foods. School as source of exposure, feelings of lack of control for parents. Safety net programmes facilitated acquisition of healthy food.
Gase *et al*., 2016^(34)^	To assess the relationship between the perceived food environment, self-efficacy and fruit and vegetable consumption	Not specified.	Los Angeles County, USA	General low-income population but large Latino group (*n* 1440); (urban)	Quantitative: cross-sectional study	Availability of healthy foods may help to increase consumption. The food environment and self- efficacy are positively related to healthy eating behaviours including healthier food selection.
Gichunge *et al.*, 2016^(82)^	To examine the association between home availability and consumption of traditional vegetables among resettled African refugees	Less than 5 years (*n* 22); 5 years or more (*n* 49)	Southeast Queensland, Australia	African refugees: Burundian, Congolese, Rwandan (*n* 71); (urban/resettled); purposive sampling	Mixed methods: quantitative and qualitative	Having a vegetable garden and a supermarket in the local neighbourhood increased likelihood of having traditional vegetables at home. Sourced traditional vegetables from a variety of sources. Preference for traditional vegetables. Barriers faced in the food environment (language, availability of traditional vegetables and transport). Children ate some specific traditional vegetables, others stopped when they moved to Australia.
Grauel and Chambers, 2014^(35)^	To examine issues of food insecurity in migrant and seasonal farmworkers (MSFW) communities in Oregon, through the lens of the food desert concept	Had lived in USA more than 5 years	Oregon’s Willamette Valley, USA	Labour camps (*n* 62 camps); farmworkers (*n* 13); (rural/labor camp)	Mixed methods: interviews and GIS mapping	MSFW experienced economic and physical barriers to food access, especially culturally appropriate foods. Long distance to stores, availability and price influenced shopping habits as well as lack of access to own or public transport. Low walkability due to distance to stores. Acquired food from alternative sources. Accessed government food assistance. Produce available but sometimes unaffordable. Due to work and family, had less time. Valued fresh food, perceived as healthier.
Hadley *et al.*, 2010^(36)^	To assess the prevalence and correlates of food insecurity in 281 refugees resettled in the USA	Had lived in the USA<5 years (on average of 48 months)	Refugees resettled in a mid-sized city in the Midwestern, USA	Recent refugees (Sierra Leone, Liberia, Ghana, Somalia, Togo and Meskhetian Turk) (*n* 281 & 39)	Mixed methods: quantitative and qualitative	Nearly half of the sample also noted difficulty in navigating the food environment “difficulty in the food environment” was associated with high food insecurity (*P* < 0 01). Household food insecurity linked to low incomes.
Hammelman, 2018^(50)^	To trace the urban foodscapes of thirty-one Latina migrant women in Washington, DC, in order to highlight the connectivity evident in their food insecurity coping strategies	Mentioned only one participant in the USA more than 20 years	Washington, D.C., USA	Latina migrant women (*n* 31); (urban); snowball sampling	Qualitative; in-depth interviews and sketch mapping	Participants were of low socio-economic status. Depended on social relationships and mobility to acquire food for their families. Barriers included cost of food, time, transport and competing needs. Visiting multiple stores to get hold of the most affordable foods that they wanted. Difficulty in accessing food benefits. Most commonly used food sources (nearby grocery stores (84 %), sharing and exchanging within social networks (52 %), Latin American groceries (52 %) and emergency distribution centers (42 %). Fresh, unprocessed, organic foods were preferred.
Hammelman, 2018^(37)^	To explore how migrant women living in poverty rely on informal networks for growing and sharing food, seek out organic, fresh foods and utilise independent survival strategies	Not clear; one participant lived more than 10 years	Colombia and Washington, DC, USA	Low-income migrant women (*n* 72); (urban)	Qualitative; in-depth (a semi-structured interviews)	Low-income women finding ways to access healthy foods, seeking out cheap stores, growing food, sharing and exchanging food in social networks and sharing transport to access foods. They wanted organic, natural, unprocessed foods. They also accessed foods through emergency food providers.
Henderson *et al*., 2017^(73)^	To explore the challenges and opportunities associated with attempting to maintain a healthy traditional diet for newcomers	From 6 months to 6 years	North End neighbourhood of Winnipeg, Canada	Newcomers (*n* 8); community workers (*n* 4); (urban); purposive sampling	Mixed methods: photovoice approach and in-depth (semi-structured interviews)	Limited access to good quality, fresh traditional food; low incomes and unaffordability of traditional foods. Access to transportation facilitated acquiring food. On social assistance or low paid jobs, unable to afford to buy enough nutritious foods. Difficulty navigating the food environment, how to decipher healthy from unhealthy in the new food environment. Abundance of processed food. Children exposed to processed foods at school and pressured parents to provide these foods at home. Time constraints meant less time for food provisioning.
Jacobus and Jalali, 2012^(57)^	How and why Lewiston’s immigrant population might be vulnerable to food insecurity and how to ameliorate this condition are important policy issues that need to be assessed in Maine.	Not specified.	Lewiston, Maine, USA	African immigrants; (community leaders); (*n* 9)	Quanlitative; in-depth interviews	Limited availability of culturally familiar products, proximity to a food source was of importance. Concerns about religious restrictions regarding halal foods. Few halal stores, expensive; preference to buy all foods from these stores, though unaffordable. To access traditional foods, personal vehicles or public transport needed.
Judelsohn *et al.*, 2017^(58)^	To explore the experiences of refugees from Burma in navigating food environments in the USA and explore the extent to which local governments are supporting or hindering their access to culturally preferred, nutritious foods	Lived in the USA for over 6 months	Buffalo, New York, USA	Burmese refugees (*n* 28, snowball sampling); Local government (*n* 7) and civil society representatives (*n* 6)	Mixed methods; qualitative, quantitative and spatial data (GIS)	Challenges to navigating the food environment: direct factors include limited transportation infrastructure, limited language skills, limited land for growing, lack of information about food safety. Indirect factors include poor-quality housing and social isolation. They continued to eat their traditional food after resettlement. Younger generations and children exposed to US foods through school. Used food assistance, gardening and foraging. Sourced food from a number of stores.
Kiptinness and Dharod, 2011^(49)^	To understand food shopping and dietary practices among Bhutanese refugees in the USA	Had lived in the USA on average 9 years	USA	Bhutanese refugees (*n* 14); (urban); snowball sampling	Qualitative; direct observation and semi structured interviews	Shop at multiple stores for food (by foot or get a ride with other Bhutanese). In USA: perceived high variety of food but more expensive. Received SNAP benefits that covered the entire food budget. Participants preferred traditional food. Used to shopping in open air markets before migrating.
Leu and Banwell, 2015^(85)^	To investigate potential dietary changes among Southeast Asian international students living in self-catered accommodation and to consider implications for their health.	Studying at the Australian National University for at least 1 year	The Australian National University, Australia	International students from Southeast Asia (*n* 31, 58 % female); urban	Qualitative (Ssemi-structured interviews)	(a) Affordability: pick the cheapest one but it depends on the quality; compared prices, (b) Accessibility: willing to take the time to travel the extra distance by bus or car to seek out a particular ingredient, (c) availability of traditional foods, but high prices limited their use. Eating out was a social event. Time scarcity increased usage of quick processed foods.
Lindsay *et al*., 2009^(38)^	To examine complex influences on immigrant Latina mothers’ feeding practices and their children’s eating and physical activity habits	Had lived in the USA for more than 3 years	The greater Boston urban metropolitan area, USA	Low-income Latina mothers (*n* 51); (urban); purposive sampling	Qualitative; focus groups and in-depth interviews	Economic constraints, food pricing and food insecurity as barriers to healthy eating. Healthy food was more expensive. Getting hold of more affordable foods by visiting discount stores, using coupons and the government WIC program. Due to work and other constraints, time for food provisioning was limited. Limited social support. Independent transport to stores to access more affordable food, lack of this transport meant accessing the local stores. High cost and low variety of healthy foods like fruits and vegetables in neighbourhood stores. Under pressure from children to provide advertised processed foods; eat out for convenience and for children.
Lofink, 2012^(87)^	To examine how aspects of a specific locality, history and set of practices interact to produce an obesogenic environment	Not specified.	East London, UK	British Bangladeshi adolescents (*n* 447); (school)	Mixed methods: quantitative and qualitative; (the ANGELO framework)	During school hours, accessed unhealthy foods outside schools; consumed the less healthy part of the school lunches. There was high availability and affordability of fast foods on their way home from school; normalised consumption of these foods. At home, traditional foods supplemented with energy dense nutrient poor foods. High access to traditional foods. Traditional foods were highly valued by parents, as well as a way to pass on traditions.
Mannion *et al*., 2014^(79)^	To understand the acceptability of a purse-sized nutrition resource and to help Sudanese refugee women identify and purchase healthy foods and navigate grocery stores.	Less than 1 year	The Margaret Chisholm Resettlement Centre, Canada	Sudanese refugee women (*n* 18); purposive sampling	Qualitative (focus groups)	Language, transportation and an unfamiliar marketplace challenged women and prevented them from exercising their customary role of ‘knowing’ which foods were ‘safe and good’ for their families. Mothers tried to feed their children foods they considered healthy; children preferred processed foods, which were sometimes provided. Relying on husband and children for navigating the food environment, children influencing the family by which foods they have been exposed to at school. Language barriers, lack of access to transport. Issues identifying packaged foods.
McElrone *et al*., 2019^(62)^	To identify the perceived dietary acculturation barriers and facilitators to food security among female Burundian and Congolese refugees	The mean length of time in the USA was 67·1 months (sd ± 47·86) ranging from 12 to 137 months	The Southeastern region of the USA	Sub-Saharan African (female Burundian and Congolese refugees *n* 18); Snowball sampling	Qualitative (semi-structured interviews)	Emerging themes: (a) difficulty with language, (b) unfamiliar cooking methods and shopping; (c) lack of public or private transportation access as a major barrier to food outlets; (d) social networks played a role in locating culturally familiar foods; (e) reliance on nutrition assistance programmes; (f) limited culturally relevant food and land access; and (g) programme policy miscomprehension.
Meierotto and Som Castellano, 2020^(39)^	To examine the various strategies that farm workers use to provide food for themselves and their families	Not specified; (moved into the area prior to the 1990s)	Head start centres, Idaho, USA	Latino farmworkers; (*n* 31); (hops farms)	Mixed methods: ethnographic observation, interview and survey	Low incomes, far distances requiring own transport as no public transport is available to larger cheaper stores. Limited time for food provisioning due to being time stretched. Double work burden for women; some help from husbands. High cost of foods limited the procurement of foods they preferred; they valued traditional foods. Food assistance schemes were utilised.
Moffat *et al*., 2017^(74)^	To investigate three pillars of food security (food availability, access and use) for immigrants and refugees living in a medium-sized city in Canada	0–5 years (*n* 15; 63 %); 6–10 years (*n* 5; 20 %); 10+ years (*n* 4; 17 %)	Hamilton city, Ontario, Canada	Mixed immigrant and refugee (*n* 24); (low-income neighbourhoods); convenience sampling	Qualitative: focus groups	Food availability: lack of availability of valued high quality, fresh, less processed, chemical-free food. Unable to find certain foods and less variety. Food access: healthier foods expensive and too little money. Difficulty shopping: having to read and understand food labels. Using food banks: quality and types of food not acceptable.
Munger *et al*., 2015^(40)^	To describe the experiences of food insecurity, structural vulnerabilities and assets for facing food insecurity	Less than 10 years	Maryland, USA	Undocumented Latino immigrants (*n* 24); (urban/enclaves); convenience sampling	Qualitative; in-depth (semi-structured interviews)	Shortage of food, lower quality food due to lack of money leading to a lack of control over food choices. Valued foods that were nutritious, fresh and unprocessed. Were not able to access the quality, variety and type they preferred. Unreliable employment. Were weary of seeking help from government, so food benefits were not well used. Social support and food exchange.
Nunnery and Dharod, 2017^(64)^	(1) To examine the socio-demographic characteristics and prevalence of food insecurity in three groups of refugees resettled in the USA; (2) to describe themes that arose as potential determinants of food insecurity for refugees; and 3) to posit a conceptual model of the potential determinants of food insecurity for refugees and how they interrelate.	Seventy percent (*n* 69) of the sample, has been resettled in the USA, for an average of 8 years	The Southeastern, USA	Refugee women: (a) Liberian (*n* 33); (b) Sudanese (*n* 22); (c) Montagnards (*n* 42)	Mixed methods; qualitative (semi-structured interviews), quantitative	On average, 70 % experienced some level of food insecurity, differences in severity between groups. Low incomes; compromised on quality and amount eaten. Difficulty in navigating the US food environment and assistance programmes (language barriers, transportation issues and an inability to do comparative shopping). Cyclical insufficiencies; bulk buying with most resources at the beginning of the month; bought processed foods and drinks for children. Grew some of their own vegetables; did not use emergency food assistance, but took advantage of school related school meal programmes.
O’Mara *et al.*, 2021^(91)^	To explore the interaction between the food environment and food procurement behaviours in the process of dietary acculturation.	One year (*n* 1); more than 15 years (*n* 15), N/A (*n* 2)	Amsterdam East and Amsterdam New-West, the Netherlands	Moroccan women (*n* 18); purposive and snowball sampling	Qualitative (in-depth interviews and a mapping exercise)	Increased availability of traditional foods over time. Most foods, both cultural and host country foods were available, though some foods needed to be halal in order to be considered available. Balancing acceptability, accessibility and affordability when buying foods. Quality, price, time and convenience were essential considerations as well. Fathers preferred traditional foods and children preferred Dutch foods. Eating out for children’s sake.
Osei-Kwasi *et al*., 2019^(88)^	To explore participants’ perceptions of social and economic factors influencing food security among Ghanaian migrants	Less than 20 years (*n* 20; 77 %); more than 20 years (*n* 6; 23 %)	Greater Manchester, UK	Ghanaian migrants (*n* 31); (urban); purposive sampling	Qualitative; in-depth nterviews	In UK foods available all year-round *v*. Ghana (seasonal). Supermarkets sell ethnic foods. Culturally acceptable foods readily available. Travel quite a distance to purchase items.
Paré *et al*., 2019^(61)^	To identify decision making in food consumption, physical activity and usage of local fruit and vegetable programmes in an urban environment	Not specified.	Midwestern city, USA	Latino participants from five focus groups (forty-four women (96 %) and two men); (urban); purposive sampling	Qualitative (focus groups); community-based participatory	The environment’s impact on decision making (a) convenience and abundance of less healthy food options in the USA; (b) access to affordable and culturally appropriate fruit and vegetable options, the kids do not like to eat F&V and like eating out (McDonald’s or Taco Bell), time constraints limiting ability to make healthier choices. Language barrier prevents awareness of food access opportunities in the city.
Park *et al.*, 2011^(41)^	To better specify the role of Latino immigrants’ beliefs about and preferences for healthy foods in linking food access to dietary patterns	Not specified.	New York City, USA	Home visit (*n* 547 families), Latina adult women (*n* 28); (urban)	Mixed methods: quantitative and qualitative	A strong preference for fresh foods and expressed strong objections to stored and packaged foods. Children dietary preferences and beliefs regarding healthy foods reflect the new food environment.
Patil *et al.*, 2010^(63)^	To contribute to the existing literature on migration health by underscoring how refugees’ daily life complexities (interactions and activities) are at work in the social production of health in the USA.	Average of 48 months	The Midwest, USA	Somali Bantu, Meskhetian Turk and Liberian refugees (*n* 175); 64 % female; (urban)	Mixed methods; Qualitative (Open-ended questions), Quantitative	(a) Changing health concerns with migration to the USA; (b) food and shopping “Fairness” in the USA; (c) diet and health: plans to address the health concerns; (d) children food preference for American foods and dietary consequences; intergenerational conflicts; (e) employment and socio-economic Impacts on diet and well-Being; (f) transport concerns and (g) social support.
Peterman *et al.*, 2013^(42)^	To describe food experiences on arrival, current food security status and examine characteristics related to food insecurity in a well-established refugee community	Had been in the USA for at least 5 years	Lowell, Massachusetts, USA	Cambodian refugee women (*n* 11), survey (*n* 150); (urban)	Mixed methods: quantitative (survey) and qualitative (focus group and semi-structured interviews)	Food availability: always enough food in the USA *v*. Cambodia and Thai refugee camp. Food insecurity was positively associated with being depressed and being widowed and negatively associated with higher income and acculturation.
Phan, and Stodolska, 2019^(43)^	To evaluate factors impacting food practices, dietary patterns and leisure among Mexican immigrants	Not specified.	Midwestern, USA	Mexican immigrants (*n* 23); (urban); snowballing sampling	Qualitative; a semi-structured interview	Elements of the new environment (availability, accessibility and affordability of food), culture beliefs regarding food and diet, psychosocial factors and taste preferences, eating out and rarely buy groceries due to the location and distance of food market.
Pineros-Leano *et al*., 2019^(69)^	To understand how Latina immigrant mothers make feeding decisions for their children.	Had been in the USA for 10 years (average)	USA	Latinas (*n* 29); Living in non-metropolitan and small metro areas; purposive sampling	Qualitative (semi-structured interviews)	Availability and food access to fresh produce easier in country of origin. Lack of fresh food and traditional foods available. Children’s preference changed after attending school. Expressed having a difficult time accessing fresh fruits and vegetables. Cultural influence of foods prepared in host country. Location of stores was important, own transport necessary to reach outlets.
Rodriguez *et al*., 2016^(75)^	To shed light on the role of the food environment in shaping food access among immigrants living in the Region of Waterloo, Ontario	0–10 years (*n* 8; 88 %); more than 10 years (*n* 1; 11 %)	The Region of Waterloo, Ontario, Canada	Immigrants (*n* 9), community stakeholders (*n* 9); (mid-size Urban); purposive sampling	Qualitative; in-depth interviews and photovoice approach	Accessibility: had good geographic access to culturally appropriate and high-quality food, affordability: the high cost of nutritious food and participants’ ability to earn an adequate income influenced what they bought. Recent immigrants arrived with little knowledge of Canadian food practices or the local food environment but relied on other immigrants for information.
Sano *et al*., 2011^(67)^	To investigate how low-income rural Latino immigrant families succeeded or failed to meet their food needs over time.	Had lived in USA <20 years	California, Michigan, Oregon and Iowa, USA	Latino Immigrant mothers (*n* 10); purposive sampling	Qualitative (case study approach)	Microsystem: immigration status gave benefits, secure/insecure employment impacted food security; skills relating to money management and money saving strategies relating to buying food linked to food security. Mesosystem: those from more well off families in the home country were more independent, compared with poorer ones that sent remittances to home country. Most food insecure group had insecure housing and little social support. Exo- and macro system factors: food assistance, both government and private was accessed, though emergency food (private, food banks) was associated with stigma. Immigration status was an issue that impacted several aspects of life.
Sastre and Haldeman, 2021^(68)^	To examine food selection factors and influence on household food selection by newcomer immigrant and refugee adolescents.	<1 year	North Carolina, USA	Newcomer youth from Southeast Asia, Middle East, Africa and Latin America/Caribbean (*n* 68); (urban)	Quantitative (semi-quantitative survey)	Access to traditional foods varied; was easy to find most traditional foods (38·2 %), full maintenance of traditional foods (33·3 %), family support by different people take turns cooking (28·4 %). Participant teens reported influencing household food selection (60 %).
Sharif *et al.*, 2017^(59)^	To assess community residents’ perceptions of corner stores to better understand what facilitates and deters patronage at these food outlets	Not specified.	East Los Angeles and Boyle Heights, USA	Latino communities (*n* 1035); (urban); randomly sampling	Quantitative; survey	Local stores were considered more expensive and of lower quality. Patronage at stores are factors related to cleanliness, provision of culturally appropriate ingredients and customer service rather than the availability of healthy food items.
Sharkey *et al*., 2012^(44)^	To examine the use of alternative food sources by Mexican- origin women from Texas Border Colonias and determine factors associated with their use	Not specified.	Texas Border Colonias, USA	Mexican-origin women (*n* 610); (border)	Qualitative; a face-to-face survey	More than 90 % strongly agreed or agreed that there was little variety in types of foods, few grocery stores or supermarkets or high food prices in their community. Relied to a large extent on pulgas for fresh fruits and vegetables. 61·3 % travelled at least 10 miles one way to purchase groceries.
Sussner *et al*., 2008^(45)^	To examine mothers’ beliefs, attitudes and practices related to early child feeding and weight	Had lived in the USA on average 9 years	The greater Boston metropolitan area, USA	Latina mothers (*n* 51); (urban); purposive sampling	Qualitative; focus groups and in-depth interviews	Social isolation exacerbated by having less social support in the USA compared with home country. Children like to consume American foods. Little time to eat three traditional meals a day, resorting to skipping meals, eating ‘on the go’, and relying on leftovers and snacks in order to ease time pressures, practices they viewed as unhealthy.
Terragni *et al.*, 2014^(2)^	To explore the early phase of dietary acculturation after migration	Less than 5 years (*n* 2), 5–10 years (*n* 6), more than 10 years (*n* 13)	Oslo, Norway	South Asian, African and Middle Eastern women (*n* 21)	Qualitative (reflective lifeworld approach)	Host country food environments (entering a supermarket where food was sold in boxes or wrapped in plastic was something completely new). Limited food choices (‘It was very difficult to find halal food. I made food without meat. I didn’t eat meat the first year’). Linguistic barriers (‘it was really difficult in the beginning not know Norwegian’). Adapted children’s lunches to host country way; accessing halal foods was important and a challenge. Not knowing what foods contained meant excluding these.
Tiedje *et al*., 2014^(65)^	To assess (1) ways of knowing about healthy eating; (2) eating practices; (3) barriers and (4) preferences for Intervention.	Had lived in USA more than 5 years	The Midwestern city, USA	Somali, Mexican, Cambodian, Sudanese (*n* 127; 16 FG); purposive sampling	Qualitative focus groups (community-based participatory)	In USA, more unhealthy foods and easy access to junk food: mostly unhealthy (frozen, cans, junk food). Children learn about healthy eating at school. Affordability: healthy foods are expensive. Cultural foods seen as both healthy and less healthy. Due to work, parents do not have time to cook, leading to consumption of convenience food.
Vahabi and Damba, 2013^(76)^	To explore immigrants’ perceived barriers in acquiring safe, nutritious and culturally appropriate food	Immigrated to Canada within the past 5 years	Toronto, Canada	Recent Latin American immigrants (*n* 70); (urban); convenience sampling	Mixed methods: quantitative and qualitative	Food values included the quality, taste and smell of some foods; this differed from their experience of foods in home countries. Lack of culturally appropriate resources. Inadequate income meant forced to rely on welfare and the main barrier to accessing adequate food. Accessibility of food outlets varied and depended on transportation cost. Limited time for grocery shopping due to work conditions.
Valdez *et al*., 2016^(52)^	To engage residents of low-resource, Latino-majority neighbourhoods in discussions of food access in a rural yet agricultural community setting, which is typically described as a food desert	Not specified.	Rural Central California, USA	Latino immigrants (*n* 158); (rural); purposively sampling	Mixed methods: quantitative (survey) and qualitative (focus groups and in-depth interviews)	Availability: good quality of fruits and vegetables due to living in an agricultural setting. Affordability: healthy food options are too expensive. Accessibility: high presence and easy access to fast food seen as the main problem.
Vasquez-Huot and Dudley, 2020^(46)^	To identify the food relief efforts that would be most beneficial to Latinos	Not specified.	North Carolina, USA	Latina women (*n* 30); (urban)	Quantitative; the open-ended questionnaire	Limited to access quality food due to lack of knowledge and transportation issues; do not have money to buy healthy foods. Home culture gets lost when families move to the USA. Employment: working multiple jobs and have limited time for meal planning or grocery shopping.
Vatanparast *et al*., 2020^(78)^	To provide a qualitative in-depth account of the status and experience of food insecurity for Syrian refugee households in Toronto and Saskatoon, Canada.	Arrived years 2015 (*n* 5, 9 %); 2016 (*n* 49, 91 %)	Toronto and Saskatoon, Canada	Syrian refugee households (*n* 54); settlement and support agencies (*n* 15); (urban)	Qualitative (semi-structured interviews)	Inconvenient locations and distance to grocery stores in host country. Lack of specific food types. Low-income (limit purchases to cheap food such as frozen, canned or prepared foods). Importance of social networks to their food security status, including friends, acquaintances on social media platforms and their sponsors. Lack of time to prepare healthy foods. Language barriers to find cultural food (Halal) while in a grocery store and issues with understanding bus schedules.
Villegas *et al*., 2018^(47)^	To understand what factors influence alterations in health behaviour, dietary patterns and food preferences post migration	Had lived in the USA on average 12 years	Illinois, USA	Latina immigrant mothers (*n* 19); (urban)	Qualitative; focus group (a semi-structured and open-ended questions)	Availability: lack of food options and condiments for flavouring dishes. Store preferences: affordability (price). Children’s preference for processed junk foods. The quality of foods and accessibility (location) were important in deciding where they would buy food.
Willis and Buck, 2007^(66)^	To examine of current dietary patterns of Dinka and Nuer refugees from Sudan to the United States prior to dental restoration and nutrition training.	Less than 5 years (55 %); more than 5 years (45 %)	The Midwestern city, USA	Eight Nuer women and 9 Nuer and 14 Dinka men (*n* 31) (Sudan)	Quantitative (24-h food intake)	Shopping habits (at US supermarkets and ethnic groceries); Children liking US foods and fast-food. Difficulty in reading food labels to know what food packages contain. Travel long distances to acquire specific food. Consumed mainly traditional food, though some ate out. Unmarried men ate a lot of convenience food. Limited time for cooking. Fathers like traditional food and children preferred American food; ate out to please children. Meat was not consumed the same way in the home country due to issues of availability, high price or not tasting right in the host country.
Wilson *et al*., 2010^(83)^	To understand how African refugees experience, perceive and interpret their food environment and how this influences their eating behaviours and food habits	Not specified.	North-West Melbourne, Australia	African immigrants (*n* 40); (suburbs)	Qualitative; Focus group and face-to-face interview (semi-structured interviews)	In Australia (foods available in all seasons) *v*. in Africa (determined by geographical locality and the seasons). In Australia: an abundance of cheap and readily available, processed, packaged and labelled food.
Yeh *et al.*, 2008^(48)^	To illuminate the barriers and facilitators to fruit and vegetables (F&V) consumption and provide suggestions for programme planners when developing future intervention programmes	Not specified.	Four North Carolina counties (rural areas) and three Connecticut counties (urban areas), USA	A diverse multi-ethnic (African American, Latino and Caucasian) (*n* 147); (rural/urban)	Qualitative; focus groups	A transition from free, homegrown F&V to high-priced produce in the supermarkets. The convenience of purchasing pre-packaged foods and the adverse impact of the media on F&V intake by promoting ‘fast-food’. Barriers to eating F&V were inaccessibility, cost and lack of time. Facilitators were family traditions, advice from a doctor and health benefits. Latinos tried to maintain traditional eating, though the types of food available were limiting. Avoided unfamiliar foods including F&V. Preference of fresh over frozen.
Yeoh *et al*., 2014^(84)^	To examine the experiences of migrants on food security in the regional area of Australia	Less than 1 year (*n* 43; 14·3 %); 1–2 years (*n* 35; 11·6 %); Over 2–3 years (*n* 38; 12·6 %); Over 3 years (*n* 185; 61·5 %)	Tasmania, Australia	Mixed immigrants, (questionnaire, *n* 301) (interview, *n* 33)	Mixed methods: Quantitative (questionnaire) and qualitative (semi-structured interviews)	Availability: 65·8 % reported easy to find traditional food ingredients affordability: food price (55·8 % expensive, 40·2 % reasonable and 4 % cheap) accessibility: 50·2 % indicated that travelled over 4 km to buy food.
Yeoh *et al*., 2014^(86)^	To investigate the experiences of food security among migrants in a regional area of Australia (Tasmania).	Less than 3 years (58 %) and more than 3 years (42 %)	Tasmania, Australia	Asian immigrants (*n* 33)	Qualitative (semi-structured interviews)	Acculturation strategies: participants were satisfied with their current food security in Tasmania, but they still encountered some challenges in the availability (culture foods), accessibility (had own transport) and affordability (high cost) of healthy and cultural food. Increased availability of cultural foods over time, though still limited variety and types. Traditional foods from certain ethnic groups was easy to get hold of and not for others. Walked or drove to acquire foods, considered it to be easy to access foods. Travelling too far for foods was not cost effective. Accessed several types of stores. Cultural foods were expensive and required skilled budgeting, especially on a low income. For those that did not have language barriers, reading English helped them understand food labels. Social networks helped with information about food, social networks and growing their own foods were ways to help them manage.
Yi *et al*., 2020^(60)^	To describe the grocery shopping patterns and behaviours of one of the largest immigrant groups in New York City, Chinese Americans	Had lived in the USA (Mean 16 years)	New York city, USA	Chinese Americans (*n* 239); (urban)	Quantitative; Survey	Type 1 shoppers prioritised proximity to places they frequented and language (product labelling, spoken by cashiers). Type 2 shoppers prioritised food quality and cleanliness, and type 3 shoppers prioritised ease and availability of items/brands they wanted to buy.
Zou, 2019^(77)^	To determine the facilitators and barriers influencing healthy eating behaviours among aged Chinese Canadians with hypertension	Had lived in the Canada (on average of 9·7 years)	Canada	Chinese Canadians (*n* 30); (urban)	Qualitative; Telephone interview	At supermarket: promotion of healthy foods, seen as safe, healthy and good price, selection of healthy items. Local market: many food options. Restaurant: no control over what consume and lack of healthy foods. Accessibility of grocery stores. Busy and no time to cook, which affects the quality of the food they eat, making it more challenging to eat healthy food.

### Collating, summarising and reporting the results

The extracted data from the results and discussion sections of the included papers were synthesised. This was done inspired by the ‘Best fit’ framework synthesis, a practical method where data are coded into *a priori* themes, as well as additional themes for data that do not fit into the framework^(23)^. We used the Analysis Grid for Environments Linked to Obesity (ANGELO) framework to interpret the data, with the following *a priori* themes: physical environment, economic environment, socio-cultural environment and political environment (see Table 2)^(24)^. The ANGELO framework is further divided into micro and macro settings, which we did not use in our analysis as the full-text reading of the selected articles revealed that there was very little on the macro scale. Additionally, where data seemed to belong to more than one of the themes, overarching themes were created. The findings relating to each theme were discussed among the team to improve trustworthiness and to reach consensus.

**Table 2:** Analysis grid for environments Linked to obesity (ANGELO framework) from Swinburn *et al.*^(24)^

Type of Environment	Scale – settings and sectors*
Physical environment	What is available? Both visible factors like food available in food outlets, schools, point of purchase information as well as less tangible factors like availability of training opportunities, access to technology and expertise.
Economic environment	What are the financial factors? Costs related to food. Costs of food production, distribution and food retailing as well as income.
Socio-cultural environment	What are the attitudes, beliefs and values related to food? Cultural norms are influenced by gender, age, ethnicity, traditions, religion, sub-group affiliation which affect behaviour of individuals.
Political environment	What are the rules related to food? Laws, regulations, policies (formal or informal) and institutional rules.

* In this review, the settings (micro) and sectors (macro) were not separated. This slightly modified ANGELO framework was used for the analysis.

## Results

**Fig. 1:**
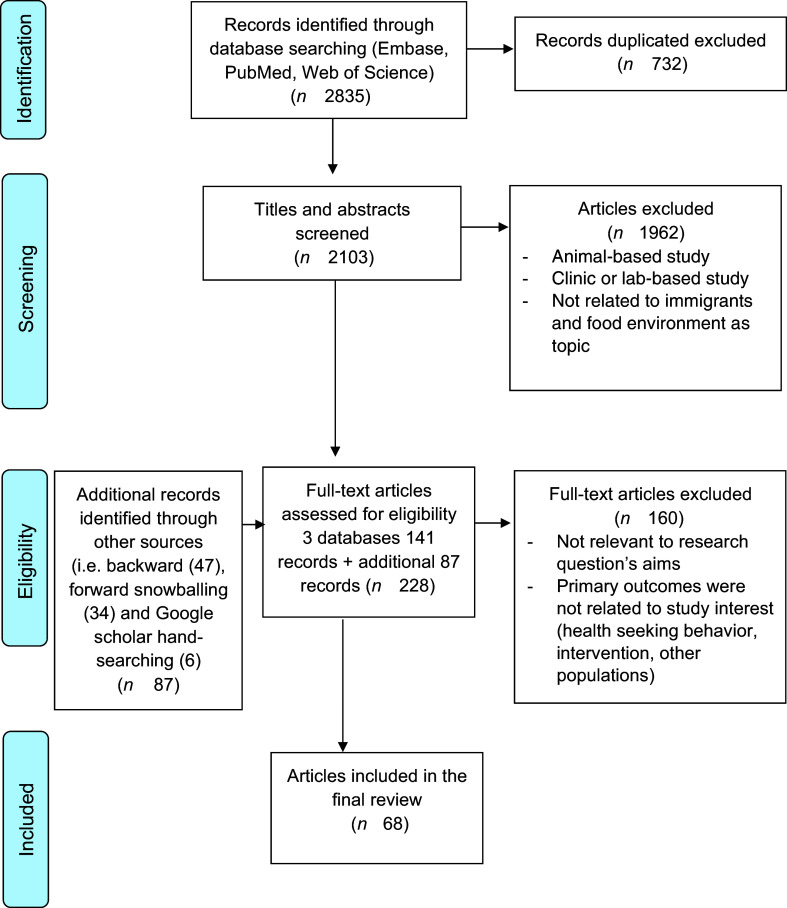
Flow diagram of literature search and selection

A total of 2835 records were identified in the initial search and after removal of duplicates, 2103 were screened for title and abstract. Backward, forward snowballing and search in google scholar identified eighty-seven additional articles. In total, 228 articles were eligible for full-text screening (see Fig. 1). Finally, a total of sixty-eight articles were eligible for inclusion. Out of sixty-eight articles, the vast majority (forty five) studied populations living in the USA^(25–69)^; ten were based in Canada^(70–79)^, seven from Australia^(80–86)^, three from the UK^(87–89)^, one from Switzerland^(90)^, one from Norway^(2)^ and one from the Netherlands^(91)^. The immigrant groups were from Asia, Africa, Middle East, South and Central America and the Caribbean. Of these, forty two were qualitative, nine were quantitative and seventeen were mixed methods studies. Around 35 % of the studies included only women and the remaining were mixed participant populations.

An overview of the sixty eight included studies is shown in Table 1, and the results are presented below according to themes from the ANGELO framework, followed by overarching themes.

### Physical environment

Out of sixty-eight included articles, fifty two had data pertaining to the physical environment.

#### Host country food environments

In the low-income neighbourhoods where immigrants resided, there was easy access to fast food outlets and unhealthy food items in stores^(30,57,61,65,87,89,91)^. Fruits and vegetables were reported as being of low quality and of limited variety in the neighbourhood stores^(28,38)^. In some areas with a high proportion of immigrants, there were ethnic stores that catered to their food preferences^(74,87)^, while others travelled greater distances either to access these or for greater variety or lower prices^(2,35,40,61,84)^. In general, immigrants living in urban areas had better access to stores, as well as cultural foods, than those in rural areas^(78)^ where access to a car was necessary^(69)^.

#### Availability of specific food types

Immigrants reported that there was an overall abundance of food in the host country^(64,70,86)^; produce was always available and not just seasonally as they were used to^(71,83,88,91)^. Eating a healthy diet based on fresh foods was challenging as they were more difficult to access, particularly in smaller metropolitan areas that depended on seasonal produce^(69)^ while neighbourhoods were filled with stores providing unhealthy options^(41,61)^. In larger cities and places where immigrants had resided over a longer period, it was easier to access cultural foods^(2,84)^ and for early arrivals from different ethnic groups it was more of a struggle^(2)^. Accessing halal foods in order to eat according to Islamic religious principles was essential for Muslim immigrants^(78,91)^. Moreover, the articles referred to an increased availability of cultural including halal foods over time^(2,85,86,91)^. In different settings, some groups had an easier time than others to find their cultural foods, though many found it a challenge^(58,61,62,68,70,73,75,76,82,86,91)^. There was a lack of familiarity and variety thereof^(86)^. Halal foods would have to be accessible for meat and meat-containing products to be considered as ‘available’ for consumption, in the same way as unfamiliar fruits and vegetables tend to be ignored^(64,78,91)^. Feeling uncertain about the content of food items, particularly in the early period, meant excluding them, reducing available options^(2)^.

#### Avenues for sourcing food

Large supermarkets were considered as the basis for most immigrants’ food shopping – a one-stop shop to buy a variety of good quality, affordable food including produce in a clean environment^(35,39,51,54,56,64,66,84,86,91)^, though some felt uncomfortable in the ‘sterile’ environment^(57)^. With demand, supermarkets increased the amounts of cultural foods they sold^(91)^. These supermarkets were often further away, requiring transportation^(29)^, and local stores were considered more expensive and of lower quality^(32,56,59)^. In order to acquire foods, participants typically visited several stores or food sources^(27,32,47,49,53,56,58,60,82)^ particularly ethnic stores to supplement what they could procure at the supermarket.

Ethnic stores were clustered in areas where immigrants resided^(51)^, and they were frequented to purchase culturally specific foods including fruits, vegetables, meat and other ingredients^(27,51,56,58,59,64,66,82,84,86)^. The ethnic shops had a personal connection with customers^(27)^ and by speaking the language of customers, helping them to understand product labelling^(60,79)^. Immigrants who had poor host country language skills, as well as those with religious dietary restrictions, preferred to rely on the ethnic stores^(57,60)^ and others lacked skills to buy foods outside of halal stores^(79)^. In this way, they could be more independent and shop on their own^(64)^. Ethnic stores were found to fill a gap in the provision of healthy food in areas deemed food deserts^(52,89)^.

Other food sources were frequented primarily for fresh produce; these included farmers’ markets^(43,46,54,56,75,82)^, Pulgas (flea markets), fruit and vegetable stands, vendors, farms, livestock markets as well as family, friends or neighbours^(29,35,44,52,58,61,75)^ or by foraging for food^(35,52,58,75)^. Many immigrants cultivated or longed to grow their own food again^(37,39,56,58,62,64,65,73)^, as a way to access tastier, more nutritious, culturally appropriate fresh produce they could trust, while saving money^(37,52,54,56,58,65,75,82,86)^.

#### School food environment

Children of immigrants were introduced to host country foods including highly processed foods through school food^(30,39,49,58,63,65,69,71,72,91)^, which also led to a change in preferences^(63,69)^. For packed lunches, mothers soon stopped sending traditional food as they came back uneaten and learnt how to make lunchboxes in the way of the host country^(2,71,91)^. Reasons for not taking traditional foods as a school lunch included lack of facilities to heat up food, not wanting to spill, food allergies, food odors, short periods to eat, and importantly, children wanting to fit in^(71–73)^.

### Economic environment

Out of sixty-eight included articles, forty had information pertaining to the economic environment.

#### Socio-economic circumstances and food access

In general, immigrants had limited incomes^(29,30,36,37,66,73,74,76)^; the first 2 years were particularly precarious, though the financial situation of immigrants improved over time^(74)^. Poverty meant that there were many competing needs and these were difficult to manage^(28,36)^. Rent and utilities needed to be prioritised over other things, such as food^(50)^, and likewise food was prioritised over health and other costs^(37)^. In addition, those with families needing support in the home countries, sent remittances, further reducing their disposable income^(67)^. More than half the monthly budget was spent at the beginning of the month on provisions^(64)^.

Low incomes were centrally linked to food insecurity^(35,36,38,49,50)^ and food decisions are majorly impacted by income^(78)^, leading to a lack of control over food choices^(86,90)^. Food prices were often high in relation to the income of immigrants^(74)^, which was a hindrance for buying food^(42,64,86)^. This was particularly relevant for nutritious food like fruits, vegetables fish and meat that were more expensive^(28,29,52,61,65,84–86,90)^. Culturally specific foods were also considered more expensive^(28,40,53,57,58,63,73–76,78,80,81,84,85)^, particularly in places where there was a low presence of these foods^(84)^. Even though halal stores were considered trustworthy, they were too expensive to rely on for all food purchases^(78)^. For Muslim immigrants, the price of halal and non-halal foods influenced the type, quantity, quality and nutritional value of foods acquired^(78)^. In addition, fruits and vegetables (and ethnic foods in some cases), were more expensive in local food stores in walkable areas and cheaper in larger stores, further away, requiring transport^(29,46,47,50,54,56,61,76,81)^. The quality of food was a major concern, where fresh foods without chemicals were desired, ‘organic’ foods were unaffordable^(58,74)^, whereas processed foods including fast food were cheap^(28,71,85,87)^. The cost of food determined where they shopped^(32)^, what they purchased and the variety they consumed^(38,55,71)^. Participants needed to buy the cheapest foods, so they found the sources with the lowest prices for the items they wanted, often good quality, fresh food^(43,47,50)^.

### Socio-cultural environment

Forty-one articles out of sixty eight included articles had data pertaining to the socio-cultural environment.

#### Food values

Food values refer to immigrants’ desire for high quality, fresh, chemical-free and unprocessed foods, in particular fruits and vegetables, natural foods in their natural state^(33,35,41,53,58,64,85,90)^, perceived as good for health^(41,43,69,75)^. Fresh and homemade foods were important to immigrants^(65)^. They regarded host country foods with suspicion and equated these foods, particularly processed, preserved, canned or frozen foods as being old, filled with chemicals and therefore unhealthy and undesirable^(37,48,61,78)^. Even the fruits and vegetables sold were viewed as having chemicals and hence there was a strong desire for organic foods^(33)^. Fruits, vegetables and meat were experienced as having less taste and fragrance as compared with their home countries^(25,31,32,43,47,70,71,74,76,78,80,85,91)^, and this was perceived as evidence of lower quality and nutritional value^(76,78)^. Some did not trust tap water for consumption and relied on bottled water^(64)^. Not knowing where foods came from led to a lack of trust.

#### (Cultural) food preferences

Overall, immigrants expressed a strong desire to eat their traditional foods^(65,76,82)^ based on fresh foods they considered healthier^(33,43,65,69,70,73,75,76,82,85)^, maintaining these eating habits was important to them^(73,82)^. However, some thought that their cultural foods were ‘greasy’ and had too big portion sizes as well as consisting of lot of meat^(65,91)^. Preparing traditional foods reinforced the link to the home country and was a way to pass on traditions^(30,57,65,82)^, whereas adopting host country foods made them feel more integrated^(65)^. Limited accessibility to preferred foods and the lack of flavour in host country foods forced them to find new ways of making traditional dishes^(43,70)^ with familiar produce^(46,56,73,82)^. To prevent dietary acculturation, some parents tried to control children’s food choices and mainly provided traditional foods at home^(69,75)^. Immigrant families varied from eating primarily traditional food to eating a combination of both traditional and host country foods^(68,85)^. Parents, particularly fathers, preferred traditional food^(63,65,66,69,79,91)^. Some immigrants living in smaller metropolitan areas adapted to what was available and served their children processed foods due to limited access to healthier foods^(69)^. The convenience of host country foods was appreciated, though perceived as having potential negative health outcomes^(70)^. In the home country, meat and packaged foods were seen as luxuries^(64)^, while they were eaten more often after migration^(63)^. Immigrants would sometimes crave unhealthy foods, both host country and traditional forms^(65)^. For those with onset or presence of a health condition, it affected how they ate and therefore procurement to some extent^(69)^. Participants from Africa, Middle East and South East Asia, most of whom were Muslim, reported that religion was very important in determining their food choices^(2,68)^.

#### Social support networks and changing roles

Immigrants’ reported relying on social networks from the same ethnic community generally and in relation to acquiring food^(50)^. The family unit was at the core of the support circle and friends and neighbours were also included^(37)^, though they experienced much less social support and connection than in their home countries^(37,45,50,78)^. Within the family resources were pooled and shared for food^(37)^ and social networks mainly supported acquisition of affordable food through transport (rides in personal vehicles) and sometimes childcare^(30,37,50,58,64)^. There was also an exchange of money, services and food with friends and other community members^(64)^. Ethnic enclaves facilitated cultural norms in the host country as well as enabled easier support through social networks^(26)^.

Although men were more involved in household chores following migration, women found themselves responsible for practically all aspects of home life, with less time for food preparation^(78)^. Cooking was a responsibility that seemed allocated to women irrespective of their employment status for the majority^(25,68)^. If there was no woman in the household available to cook, ready meals and convenience foods were more likely to be relied upon^(25)^. Food went from being a social aspect of life to fulfilling more of a biological function^(45)^.

#### Children’s influence

Children were more acculturated through exposure to outside food environments like school, neighbourhoods and peers as compared with their parents^(33,47,58,71,73,81,82)^. They had a preference for and wanted parents to provide host country foods, often processed ones^(30,47,58,66,71,79,91)^. Parents wanted to provide what they knew as good and healthy food, which were often rejected by their children^(33,45,61,71,82)^ and some started cooking host country foods for the whole family, in spite of protests from their husbands^(91)^. Prioritising family cohesion and positive relationships meant providing the desired foods^(30,66)^. There was a conflict between the food parents valued and what children desired^(30,41,82)^, wanting them to eat a sufficient amount^(30)^ and being happy^(63,91)^. Some parents looked for acceptable (halal) versions of fast foods^(63)^ and learnt how to make the host country foods that the children asked for^(69,79)^. In this way, children were agents structuring shopping and dietary intake^(63)^. Some parents who had experienced food shortages compensated by letting children indulge in foods of their choice^(64,69)^. In a recent study on adolescents, 60 % reported that they influenced household food selection and 21·5 % reported having full control over what was eaten at home^(68)^.

#### Eating out

Pre-migration, eating out at restaurants was an occasional treat^(43)^; however, after migration eating out became much more common, so much so that it became a regular event even for working class immigrants^(43,61,70,87)^. Some immigrants found themselves time poor and eating out or consuming convenience food was a way to have something to eat that was cheap and fast, replacing to a certain extent the burden of shopping and cooking foods at home^(43,85)^. Fast food restaurants were experienced as cheap and child friendly, facilitating eating out as families^(38)^, though it took away the control over healthier choices^(77)^. Families ate out more due to children’s desire for fast foods^(66,91)^.

### Political environment

Twenty four out of sixty-eight included articles had content pertaining to the political environment. Primarily, government support and food assistance programmes were mentioned. The use of such benefits and assistance was related to need, awareness, cultural norms, past experiences and language barriers^(66)^.

#### Government food related benefits

Government food-related benefits, such as Women, Infants and Children, Supplemental Nutrition Assistance Program and free and reduced school meals in the USA and the Australian Centrelink were all mentioned in the studies and enabled families to have enough food till the end of the month. Food benefits were highly depended on^(64)^. This was particularly appreciated in times of need^(38,58)^ and was seen as a facilitator to food security^(62,67,86)^. Though these benefits were supplementary in nature, many families reported them as the main family food budget^(27,37,39,49,62)^, although insufficient^(28,29,53,63)^. However, not all immigrants accessed all benefits^(67)^. The knowledge, time and resources needed to apply for state food benefits, particularly relating to automated and literacy demanding application processes, prevented some immigrants from applying^(28,33,39,50,64)^ or reapplying once they lapsed^(62,64)^. Undocumented migrants or others with concerns about their immigration status may also be deterred from accessing these schemes^(29,39)^.

These safety net programmes helped immigrants access healthy food and improved access to culturally acceptable, staple foods^(30,33,37–39,53,58)^. Benefits were typically spent within 1–2 weeks of issuance^(27,49)^. Immigrants then resorted to cheaper food including host country foods^(53)^.

#### Food assistance

Immigrants mentioned having used emergency food assistance (food pantries, food banks) in the past, particularly in their first 2 years in the host country^(74)^ and from being very reliant to not using it at all^(53,64)^. Faith-based organisations providing food assistance were perceived as safe regarding immigration status since they did not require identification^(40,76)^. Barriers to usage included stigma, issues of access^(40,76)^ as well as food being of poor quality or culturally inappropriate, like canned food^(74,76,78)^. For Muslims, food assistance was often inappropriate since they did not provide halal foods, resulting in wastage^(78)^. Food pantries provided standard sized packs of short term emergency food relief^(28,29)^ and relied on donated items, often with a long shelf life^(39,73)^. There was very little fresh food and what was available was often old or rationed out quickly^(76)^.

### Overarching themes: interconnectedness between aspects of the food environment

In addition to the four distinct themes based on the ANGELO framework, we identified three themes that characterised the interconnectedness between different aspects of the food environment interactions and immigrant populations: time scarcity (sxiteen articles), mobility (twenty-six articles) and navigating the food environment (forty-four articles).

#### Time scarcity

Available time, primarily linked to gender based double work burden, played an important role in determining the extent to which immigrants could pursue food provisioning activities and therefore in which way they interacted with the food environment. This theme was a combination of the socio-cultural, economic and physical environments. Life following migration was described as hectic and time was scarce due to women being engaged in paid work, studies or other commitments, while continuing to be responsible for caring and preparing food for the family^(32,39)^. For those in paid work, time scarcity was a major issue; there were often long hours^(80)^, multiple jobs and long distances to travel to work, including sometimes working at night to care for children during the day^(29)^. For some this meant ending work late when most food stores were closed, apart from corner stores that sold limited healthy options^(28,29)^. These structural changes within the family shifted the eating patterns of the whole family^(45,61,71)^. Lack of time as well as childcare responsibilities minimised time for shopping, making it more challenging to prioritise healthy foods and cooking from scratch^(32,39,46,73,80)^. Not having enough time meant that food provisioning needed to be easy, fast, convenient and close by^(91)^. This sometimes led to time-saving shortcuts, including turning to and becoming reliant on convenience foods, leftovers, snacks, skipping meals or eating on the go, something they were aware was not conducive to their health^(45,61,63,71,77,78)^. Cheap processed foods were used during time scarcity since traditional foods took longer to make^(65,85)^. However, foreign-born women were more likely to view food provisioning as an essential task as opposed to weighing in the effort required when buying and preparing food^(32)^.

#### Mobility

Being flexible about where to buy foods allowed access to more affordable foods that aligned with their values and preferences and therefore determined how immigrants interacted with their food environment. Money and time constraints were compounded by lack of transport^(63)^. This theme could be seen as an interplay of the physical, socio-cultural and economic environments, as well as the previous theme, time scarcity. Being mobile was a way of trying to reduce food insecurity^(50)^. Access to transport and time therefore facilitated this process by allowing for the acquisition of healthier affordable foods, by being able to travel further and to travel to multiple stores that offered the food they wanted, at prices they could afford^(28,38,50,81,82,85)^. Proximity of food shops to home was one factor in determining access^(86)^. Owning a car or relying on family and social networks within the larger ethnic group to acquire rides were key^(44,49,50,58,62,63,86)^. Public transport routes and timings were limited for those who lived further away from the center^(57)^, costing money and time, with inconvenient connections between neighbourhoods and food stores^(64,85)^. Walking or relying on public transport meant carrying multiple heavy bags and quantities purchased were limited to what they could carry themselves^(62,63,82)^. Additionally, being accompanied by children and walking distances^(32,38,50,73,82)^; this meant visiting fewer stores and some food sources were not accessible at all^(63,82)^. Weather conditions and cold season were an added challenge when relying on public transport^(62,78)^. Some women were dependent on others since they did not know their address, and others could not travel by taxi due to religious restrictions for women^(79)^. Some could not afford cars, while others acquired personal vehicles as soon as they were able to, in order to facilitate food procurement^(57,58,62,85)^. The high financial costs of car expenses^(78)^ meant weighing whether it was worth travelling further for the amount saved in cheaper food^(86)^. Cycling was mentioned by international students who pushed their cycles home loaded with groceries^(85)^. Those who relied on ethnic stores for most purchases travelled further to more affordable stores^(60,66,78)^. Immigrants were willing to travel through the city or beyond for food that was suitable in relation to cost, quality, what was valued and cultural preferences^(43,46,51,62,78,81,82,84,85,88)^.

#### Navigation

This theme combined aspects of the economic environment, socio-cultural and physical environment with themes of time scarcity and mobility. Immigrants often faced a new language and a new food system^(35)^ and relied on members of their community to initially guide them in the new food environment^(49,62,63,75,78,79,86,90)^ including shops, products and new ways of eating^(2)^. Neighbours from the same religion showed them how to identify and where to get hold of appropriate foods^(62,78)^. Social media groups shared how to access and determine culturally appropriate and affordable foods, including halal foods and current deals^(78,86)^. Without this, difficulties accessing foods and stores were harder to overcome^(58,63)^. Yet, navigation improved with time; it took the first few years to confidently shop for foods^(2,75,76)^. Pre-migration food procurement skills included acquiring quality raw, fresh foods from markets, stores or home gardens^(35,63,73,82,83)^ and now foods were in unfamiliar packaging and methods of storage, such as frozen foods^(2,66,74)^. Those with little prior experience of food provisioning before migration or were cooking for themselves acquired more easy convenient food^(66)^.

Lack of language skills and literacy were barriers to food security^(62,67,78)^, navigating public transport^(58,64)^, identifying stores, food items and deals on food^(36,64,76)^ or being able to read and understand food labels^(2,58,61,63,64,73,78,79,86,90)^. For some, language barriers persisted over time, particularly for older immigrant women^(62)^, making it harder to be independent in procuring food^(79)^. For Muslim immigrants, there was a fear of not adhering to halal standards, which meant restricted options^(2,78)^. This meant that some had to shop with their husbands or children^(64,86)^, and therefore children had to tag along, indirectly leading to more processed foods and sweet items being bought^(63,69,79)^. Some believed that if food was for sale in stores, it must be healthy^(79)^. For some, there was a lack of trust even towards ‘halal’ foods as there had been cases of foods deliberately mislabelled as halal^(2)^. In other groups, women were better at navigating the food environment than men^(66,84)^ and those who migrated from urban environments found it easier to adapt to the new food environment than those from rural areas ^(74)^. Physical access (location and transport) was a deciding factor in where participants purchased their food, facilitated by social networks^(49)^. Self-efficacy also a played a role in perceived ease of access to fruits and vegetables^(34)^.

Immigrants implemented a range of strategies in order to feed their families, which spanned across all the themes. Overall, they aimed for the best quality at the lowest price at the most convenient location^(91)^. Which strategies were used depended on a variety of factors such as access to time and money, availability of cheap food and transport^(37)^, a working knowledge of the local language^(27)^ and social networks. There was a cyclical pattern of having enough at the beginning of the month and having a shortage at the end of the month, when staples were relied on^(64)^. The use of coping strategies that included a variety of activities to take advantage of deals^(32,37,38,43,50,54,55,84)^, aimed at getting cheaper but healthier food^(37,38,50,54,55,84)^, was associated with being more food secure^(67)^. Shopping for fruits and vegetables meant being flexible and taking advantage of deals and seasonal foods^(38,47,54)^. Time-poor immigrants particularly relied on stretching their budget^(55)^ by buying inexpensive staple foods in bulk to last the month^(27,30,37,40,49,64,76,86)^, non-perishable items to stock up on^(27,30,33)^ and cooking cheap traditional meals^(49,64)^. Due to financial constraints, participants reported compromising on the variety^(37,50,64)^ and quality of food in order to have a sufficient amount to eat^(40,53)^. More expensive food items were adjusted by decreasing the amount bought^(86)^. Foods were prioritised in different ways, such as foods higher in protein and foods that do not spoil easily or are the most filling^(50)^. Limiting the purchase of more expensive foods, supplementing with homegrown food^(27,37)^ and eating at home also helped to lower spending^(30,33)^. Social networks sometimes functioned as a place for food sharing as well as buying prepared foods from neighbours or friends^(37,39,44,50)^. They turned to frozen, canned and prepared foods to deal with economic access^(78,85)^. When money was finished, food was sometimes bought on credit at ethnic stores^(64)^. Skipping meals, eating cheap processed foods and as well as kids taking advantage of food at school were coping strategies^(64)^. Cheap fast food allowed families to eat while on a budget^(65)^. To afford fruits and vegetables, they frequented market stalls or Pulgas^(52)^, as well as buying foods on sale^(50)^ or buying seasonal foods^(47)^. When the budget was tight, quantity was prioritised over quality^(25,46,90)^. Fresh produce was weighed against more satiating higher energy foods such as fast food and meat when making decisions based on a limited budget^(54)^. Fresh items were purchased and consumed more towards the first half of the month^(63)^. Some reported reducing vegetables and meat and relying more on cheap culturally appropriate food^(78)^. Some ethnic groups seemed to manage on what they had, whereas others struggled to have enough at the end of the month^(63)^. Overall, the process was time intensive and required complex decision making and prioritising in order to make the whole effort worthwhile^(37)^.

## Discussion

In this scoping review, we identified sixty-eight studies addressing immigrants and the food environment published between 2007 and 2021. There was a paucity of research from countries other than the USA and a strong focus on women. Our major findings focus on the interactions between immigrant consumers with different aspects of the food environment and their interconnectedness: (1) Fresh high-quality natural foods and cultural foods were strongly valued, though children were more exposed to and demanded host country (often nutrient poor) foods; (2) Navigating the new food environment on a low income resulted in coping strategies where additional food skills were needed and (3) Time and mobility were key to determining potential trajectories of accessing healthier or less healthy foods.

Immigrants valued fresh, chemical-free, unprocessed healthy foods and had a set of skills and strategies to buy and prepare these, in spite of living on low incomes and facing other barriers. These values seem to be an internal motivator compelling them to surpass barriers and acquire healthier foods, stemming from a cultural or traditional discourse where simple and natural foods is deep rooted^(92)^. Several studies have found that immigrants or less acculturated groups in high-income countries are able to acquire a healthier diet at a lower cost than the host population^(93–95)^, a phenomenon termed as ‘nutrition resilience’^(95)^. In our study, this was shown in the industrious way they strived to access food they valued. This relates to findings demonstrating that availability in the environment and outcome behaviour are often not directly linked, but rather that the interaction is moderated by personal factors^(96)^. A systematic mapping review on factors influencing dietary behaviour in immigrants and ethnic minorities living in Europe had similar findings as our review, but focused more on the individual level^(97)^. Food advertising, another known influencer of food choices^(98)^, was not mentioned in our studies. It is, however, likely that parents were indirectly influenced by their children’s exposure to advertisements.

Our review characterised immigrants as struggling financially. Due to the high reported costs of cultural and fresh healthy foods, they had to compromise on the quality of food in order to have enough. This was confirmed in a study that reported high costs leading to prioritising quantity over quality, therefore limiting access to fresh foods such as fruits, vegetables, fish and meat^(99)^. Navigating their new food environment required food literacy in addition to the food skills that they had in order to enable healthier choices when buying packaged foods^(100)^. An Australian study suggested that food literacy would not remove the wider environmental and economic causes of food insecurity but could decrease vulnerability to the obesogenic environment^(100)^. Navigating this was easier if they were able to access a social network (community of the same ethnicity) that could partially bypass other barriers such as showing them where to shop or what to buy^(90)^, thus making them less vulnerable. In our study, all packaged, processed and preserved foods were grouped into foods that were not fresh and therefore less healthy. A study on perceptions of processed foods among low-income and immigrant parents confirmed these findings – where packaged food including frozen and canned foods were considered processed irrespective of the contents^(101)^. This is not just an issue of knowing how to read food labels, but rather a food value that may be a hindrance to eating well in host countries, since frozen healthy foods including vegetables may be more affordable with practically the same nutritional value as the fresh versions. Also these parents bought processed foods because their children liked them, but they did not think that these foods were as healthy as fresh, homemade foods were^(101)^, indicating that solely nutrition education may not be the most appropriate approach in order to improve immigrants’ diet.

When there was a lack of income, time and mobility were buffers to food insecurity by allowing access to affordable valued foods, as confirmed in another study^(102)^. In our review, lack of time stemmed primarily from women’s double work burden, confirmed by another study that showed immigrants, had a higher chance of being severely poor in both time and income^(103)^, and those who were employed had younger children or were single parents were more likely to be time poor^(102)^, as we found. Time scarcity seems to have an immediate effect on food choices – linked to eating out and excess energy intake, as well as a decrease in fruit and vegetable consumption^(104)^. A study on low-income women found that nutritional value became less prioritised when food needed to be put on the table quickly; however, higher levels of confidence in food preparation and cooking skills enabled them to prioritize and make more time for cooking^(105)^. Though the immigrants in our review were vulnerable in their new circumstances, they also had strong food provisioning skills and reported striving to access fresh, healthy food. A study on non-immigrants showed how food decisions were made weighing in time and monetary costs, as well as quality and health benefits of foods against time and effort^(106)^. In our review, culturally valued foods, quality of foods and monetary costs seemed to weigh more than time and effort, linked to how food provisioning may be considered an essential task.

With respect to mobility, another study confirmed our findings showing that acquiring rides was found to be convenient for purchasing larger quantities at stores that were less accessible by foot, procuring higher quality foods that aligned with their values and preferences, and at the same time, avoiding the costs of car ownership^(102)^. A study from Australia found that having access to independent transport was the key to accessing foods, rather than whether they lived in a food desert or not; this confirmed our findings on how reliance on public transport poses difficulties for food shopping^(107)^. Being mobile meant that they did not have to be confined to accessing foods solely in local stores, which were considered to be more expensive as well as perceived to have foods of lower quality.

Low- and middle-income countries are also experiencing changes to their food environments and diets due to globalisation^(108)^, which means that some changes towards a Western diet may already take place in the home country, before migration. Within country migration from rural to urban areas has also been found to cause similar changes including consumption of cheaper types, more sugar and dairy products and more meals outside of the home due to the low incomes, high food costs and lack of time in their hectic city life^(109)^. Dietary acculturation is a dynamic, multi-dimensional and complex process^(110)^ that progresses over time. Even though in our study we see immigrants striving to maintain their food culture in different ways, it represents only a part of a broader process^(111)^. Studies have shown that over time immigrants incorporate their host food cultures across a spectrum from subtle and explicit ways^(112)^. Our study also shows that the process of dietary acculturation is not so much an active choice solely due to changes in preferences after exposure to a new food culture, but rather due to an array of factors often out of their control, that interplay to push food decisions closer to that of their host country.

We found that a majority of the studies mentioned insufficient incomes in the different immigrant groups and the implications of this on different aspects of life, including food procurement. In order to access valued and preferred foods, immigrants reported travelling to stores to mitigate food insecurity. However, this implied a decrease in the budget by having to spend on public transport or fuel. Increased time spent travelling could potentially lead to less time for preparing and cooking, which may result in more reliance on convenience foods. Financial constraints paired with problems navigating the food environment, particularly in the early period following migration, may act as a catalyst of change that can be difficult to reverse, even though the aggravating factors may improve over time. This review also highlighted how unhealthy food exposure, primarily through schools and peers, has a ripple effect on family procurement and consumption patterns through changes in children’s food preferences, serving as a possible catalyst for dietary acculturation. The complex interactions inherent in this process could result in food insecurity and a diet that is less healthy, increasing the risk of obesity, type 2 diabetes and CVD^(113)^. The disproportionately high rates of non-communicable diseases including obesity among immigrants, both adults and children, is also a reflection of the cumulative effects of such changes over time^(114)^.

### Strengths and limitations

We sought to characterise interactions with the food environment in a diverse group of immigrants through studies of different designs and focus. Most studies were about Latino immigrants in the USA, hence, affecting the transferability of the findings. Moreover, the experiences of men were lacking in the literature, creating a women bias. Through the search strategy, some relevant articles may have been missed, though we covered 14 years of published research in the field. Our decision to perform an additional qualitative analysis of extracted data was based on the recommendation by Levac *et al.*, 2010^(19)^, though we chose to stay closer to the data through the ‘Best fit’ framework synthesis and prevented a ‘pressing in’ of the data by allowing ‘left over’ data to be analysed outside of the framework. The ANGELO framework was used as it explicitly links health aspects to the food environment.

## Conclusion

This study brought together evidence from a range of studies on interactions between immigrant populations and the food environment, using the four *a priori* themes from the ANGELO framework including the physical, economic, socio-cultural and political environments. Additionally, we identified the overarching themes of time scarcity, mobility and navigation that illustrated these interactions and interconnected the different aspects of the food environment. Immigrants tried to access fresh, traditional, healthier food and were compelled to do so, though they faced structural and family-level barriers that affected the healthiness of acquired food. Our study points towards the need for further research on different types of immigrant groups, including asylum seekers and refugees; families *v*. single individuals; the perspective of men; other parts of the world other than the USA that have experienced big waves of migration and the interaction of values with objective measures of the food environment. More importantly, research needs to focus on the most vulnerable and how they can be protected and supported through this process. Understanding the food environment and interactions therein is key to proposing interventions and policies that can potentially impact the most vulnerable.

## Supplementary material

For supplementary material accompanying this paper visit https://doi.org/10.1017/S1368980021003943. click here to view supplementary material
