# Food environment interactions after migration: a scoping review on low- and middle-income country immigrants in high-income countries – ERRATUM

**DOI:** 10.1017/S136898002100433X

**Published:** 2022-01

**Authors:** Aravinda Berggreen-Clausen, Sai Hseing Pha, Helle Mölsted Alvesson, Agneta Andersson, Meena Daivadanam

The above listed article was originally published without its accompanying Graphical Abstract. This has since been added to the HTML version.

Cambridge University Press apologise for this error.
