# Supplementary material for: Food environment interactions after migration: a scoping review on low- and middle-income country immigrants in high-income countries
Source: Public Health Nutr. 2021 Sep 13;25(1):136–58. doi: 10.1017/S1368980021003943 (PMC8825972; doi:10.1017/S1368980021003943)
Supplement: Supplementary file 1 [file S1368980021003943sup001.docx]

**Supplementary material:**

**Supplementary Table 1.** Search string used for the literature search

| **Database** | **Search string** | **Result** | **Search period** | **Limits** |
| --- | --- | --- | --- | --- |
| Web of Science | ALL FIELDS: (immigra* OR migra* OR emigra* OR diaspora*) AND ALL FIELDS: (diet* OR food* OR “food choice” OR “dietary change” OR “changing diet” OR “dietary acculturation”) AND ALL FIELDS: ("food environment*" OR foodscape* OR "food access" OR "food availability" OR "food store" OR "food retail" OR "food outlet" OR "food bank" OR "food shopping" OR grocer* OR "convenience store*" OR supermarket* OR restaurant* OR "online shopping" OR "food shopping" OR neighbo* OR resident* OR "urban area*" OR "rural area*" OR "local area*") NOT ALL FIELDS: (bird* OR animal* OR virus OR bacteri* OR songbird* OR mice* OR fish* OR migratory) Timespan: 2007-2020. Indexes: SCI-EXPANDED, SSCI, A&HCI, CPCI-S, CPCI-SSH, ESCI | 1552 | 2007-2021 | Human and English language |
| Embase | (immigra* OR migra* OR emigra* OR diaspora*) AND (diet* OR food* OR 'food choice' OR 'dietary change' OR 'changing diet' OR 'dietary acculturation') AND ('food environment*' OR foodscape* OR 'food access' OR 'food availability' OR 'food store' OR 'food retail' OR 'food outlet' OR 'food bank' OR grocer* OR 'convenience store*' OR supermarket* OR restaurant* OR 'online shopping' OR 'food shopping' OR neighbo* OR resident* OR 'urban area*' OR 'rural area*' OR 'local area*') NOT (bird* OR animal* OR virus OR bacteri* OR songbird* OR mice* OR fish* OR migratory) AND [1-1-2007]/sd NOT [20-12-2020]/sd | 851 | 2007/01/01 to 2021/05/14 | Human and English language |
| PubMed | (((((immigra* OR migra* OR emigra* OR diaspora*)) AND (diet* OR food* OR “food choice” OR “dietary change” OR “changing diet” OR “dietary acculturation”)) AND ("food environment*" OR foodscape* OR "food access" OR "food availability" OR "food store" OR "food retail" OR "food outlet" OR "food bank" OR "food shopping" OR grocer* OR "convenience store*" OR supermarket* OR restaurant* OR "online shopping" OR "food shopping" OR neighbo* OR resident* OR "urban area*" OR "rural area*" OR "local area*")) NOT (bird* OR animal* OR virus OR bacteri* OR songbird* OR mice* OR fish* OR migratory)) AND ("2007/01/01"[Date - Publication] : "2020/12/20"[Date - Publication]) | 432 | 2007/01/01 to 2021/05/14 | Human and English language |
|  | Google Scholar search string  We hand-searched searched the first ten pages of Google scholar with different simplified search strings and used cited function of Google Scholar for forward-searched to identify potential relevant studies. | | | |
| Google Scholar | ALL FIELDS: (immigra* OR migra* OR emigra* OR diaspora*) AND ALL FIELDS: (diet* OR food* OR “food choice” OR “dietary change” OR “changing diet” OR “dietary acculturation”) AND ALL FIELDS: ("food environment*" OR foodscape* OR "food access" OR "food availability" OR "food store" OR "food retail" OR "food outlet" OR "food bank" OR "food shopping" OR grocer* OR "convenience store*" OR supermarket* OR restaurant* OR "online shopping" OR "food shopping" OR neighbo* OR resident* OR "urban area*" OR "rural area*" OR "local area*") NOT ALL FIELDS: (bird* OR animal* OR virus OR bacteri* OR songbird* OR mice* OR fish* OR migratory) Timespan: 2007-2020. Indexes: SCI-EXPANDED, SSCI, A&HCI, CPCI-S, CPCI-SSH, ESCI. |  | 2007-2020 | The first ten pages |
| Google Scholar | ALL FIELDS: (immigra* OR migra* OR emigra* OR diaspora*) AND ALL FIELDS: (diet* OR food* OR “food choice” OR “dietary change” OR “changing diet” OR “dietary acculturation”) AND ALL FIELDS: ("food environment*" OR foodscape* OR "food access" OR "food availability" OR "food store" OR "food retail" OR "food outlet" OR "food bank" OR "food shopping" OR grocer* OR "convenience store*" OR supermarket* OR restaurant* OR "online shopping" OR "food shopping" OR neighbo* OR resident* OR "urban area*" OR "rural area*" OR "local area*") NOT ALL FIELDS: (bird* OR animal* OR virus OR bacteri* OR songbird* OR mice* OR fish* OR migratory) Timespan: 2007-2020. Indexes: SCI-EXPANDED, SSCI, A&HCI, CPCI-S, CPCI-SSH, ESCI. |  | 2020 | The first ten pages |
| Google Scholar | immigra* OR migra* OR emigra* OR diaspora* AND "food environment" OR "food access* |  | 2007-2020 | The first ten pages |
| Google Scholar | immigra* OR migra* OR emigra* OR diaspora* AND "food environment" OR "food access* |  | 2019 | The first ten pages |
| Google Scholar | immigra* OR migra* OR emigra* OR diaspora* AND "food environment" OR "food access* |  | 2020 | The first ten pages |

**Supplementary Table 2.** Inclusion and exclusion criteria for articles

| **Inclusion criteria:** | **Exclusion criteria:** |
| --- | --- |
| Immigrants from low- and middle-income countries living in host countries  Food related aspects of the food environment | Interventions  Main focus on diet or health outcomes  Built environment or food stores that did not relate to food aspects  Food security without it being reported in its components  Business aspects of ethnic food stores or restaurants  Ethnic stores relating to food aspects |
